# Root canal irrigants

**DOI:** 10.4103/0972-0707.73378

**Published:** 2010

**Authors:** Deivanayagam Kandaswamy, Nagendrababu Venkateshbabu

**Affiliations:** Journal of Conservative Dentistry Editor (2005-2007), Department of Conservative dentistry and Endodontics, Faculty of Dental Sciences, Sri Ramachandra University, Porur - 116, Chennai, Tamil Nadu, India

**Keywords:** Root canal irrigants, endodotic irrigants, NaOCl, EDTA, MTAD, CHX, *E faecalis*

## Abstract

Successful root canal therapy relies on the combination of proper instrumentation, irrigation, and obturation of the root canal. Of these three essential steps of root canal therapy, irrigation of the root canal is the most important determinant in the healing of the periapical tissues. The primary endodontic treatment goal must thus be to optimize root canal disinfection and to prevent reinfection. In this review of the literature, various irrigants and the interactions between irrigants are discussed. We performed a Medline search for English-language papers published untill July 2010. The keywords used were ‘root canal irrigants’ and ‘endodontic irrigants.’ The reference lists of each article were manually checked for additional articles of relevance.

## INTRODUCTION

Bacteria have long been recognized as the primary etiologic factors in the development of pulp and periapical lesions.[1–3] Successful root canal therapy depends on thorough chemomechanical debridement of pulpal tissue, dentin debris, and infective microorganisms. Irrigants can augment mechanical debridement by flushing out debris, dissolving tissue, and disinfecting the root canal system. Chemical debridement is especially needed for teeth with complex internal anatomy such as fins or other irregularities that might be missed by instrumentation.[4] For this review article we performed a Medline search for all English-language articles published till July 2010. We used the keywords ‘root canal irrigants’ and ‘endodontic irrigants.’

## IDEAL REQUIREMENTS OF ROOT CANAL IRRIGANTS[5]

1. Broad antimicrobial spectrum
2. High efficacy against anaerobic and facultative microorganisms organized in biofilms
3. Ability to dissolve necrotic pulp tissue remnants
4. Ability to inactivate endotoxin
5. Ability to prevent the formation of a smear layer during instrumentation or to dissolve the latter once it has formed.
6. Systemically nontoxic when they come in contact with vital tissues, noncaustic to periodontal tissues, and with little potential to cause an anaphylactic reaction.

## CLASSIFICATION

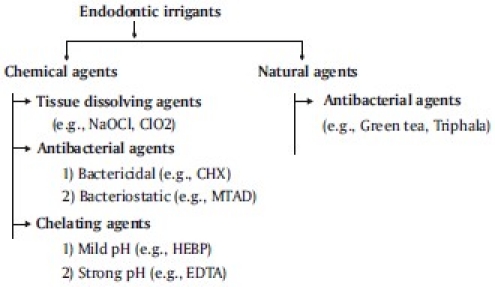



## SODIUM HYPOCHLORITE

### History

Sodium Hypochlorite (NaOCl) has an extensive history in medicine and dentistry and continues to be popular even today. During World War I, the chemist Henry Drysdale Dakin and the surgeon Alexis Carrel extended the use of buffered 0.5% NaOCl solution to the irrigation of infected wounds.[6]

### Mechanism of action

Pécora *et al*.[7] reported that NaOCl exhibits a dynamic balance as is shown by the reaction:

$$\mathrm{NaOCl}\;+\;H_{2}O\;↔\;\mathrm{NaOH}\;+\;\mathrm{HOCl}\;↔\;{\mathrm{Na}}^{+}\;+\;{\mathrm{OH}}^{–}\;+\;H^{+}\;+\;{\mathrm{OCl}}^{–}$$

NaOCl + H_2_O ↔ NaOH + HOCl ↔ Na^+^ + OH^-^ + H^+^ + OCl^-^

**Scheme 1:**
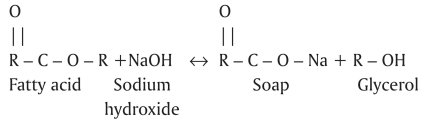
Saponification reaction

**Scheme 2:**
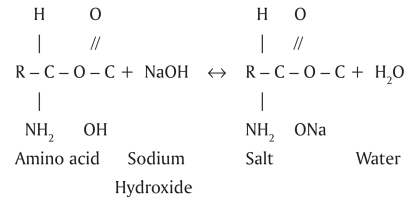
Amino acid neutralization reaction

**Scheme 3:**
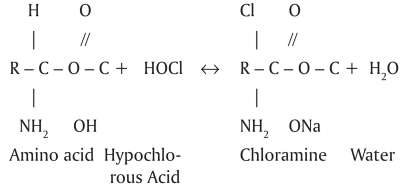
Chloramination reaction

The chemical reactions between organic tissue[7–8] and NaOCl are shown in Schemes 1–3:

NaOCl acts as an organic and fat solvent, degrading fatty acids and transforming them into fatty acid salts (soap) and glycerol (alcohol), which reduces the surface tension of the solution [Scheme 1].[9]

NaOCl neutralizes amino acids forming water and salt [Scheme 2]. With the exit of hydroxyl ions, there is a reduction of pH.

When hypochlorous acid, a substance present in NaOCl solution, comes in contact with organic tissue it acts as a solvent and releases chlorine, which combines with the protein amino group to form chloramines [Scheme 3]. Hypochlorous acid (HOCl^-^) and hypochlorite ions (OCl^-^) lead to amino acid degradation and hydrolysis.[9] The chloramination reaction between chlorine and the amino group (NH) forms chloramines that interfere in cell metabolism. Chlorine (a strong oxidant) has an antimicrobial action, inhibiting bacterial enzymes and leading to an irreversible oxidation of SH groups (sulphydryl group) of essential bacterial enzymes.[9]

Thus, the saponification, amino acid neutralization, and chloramination reactions that occur in the presence of microorganisms and organic tissue lead to the antimicrobial effect and tissue dissolution process.[9]

### Antimicrobial property – Concentration and time

The most effective irrigation regimen is reported to be 5.25% at 40 min;[10] irrigation with 1.3% and 2.5% NaOCl for this same time interval is ineffective in removing *E faecalis* from infected dentin cylinders.[11] NaOCl was moderately effective against bacteria but less effective against endotoxins in root canal infection.[12]

### Effect on biofilm[13]

1. Complete dissolution of cells with absence of visual evidence
2. Bacterial cells are disrupted and separated from the biofilm and are nonviable
3. Bacterial cells remain adherent within the biofilm but are nonviable
4. Bacterial cells are disrupted and separated from the biofilm but are viable
5. Bacterial cells remain adherent within the biofilm and are still viable.[14]

### Increasing the efficacy of NaOCl

#### 1. Altering the pH:

a. The antibacterial properties and tissue-dissolving properties of 5.25% NaOCl decrease when it is diluted.[14–16] When NaOCl is added to water, the following reaction takes place:

(1) $$\mathrm{NaOCl}\;+\;H_{2}O\;\to \;\mathrm{NaOH}\;+\;\mathrm{HOCl}\;\left(\mathrm{hypocholorous}\;\mathrm{acid}\right)$$

In aqueous solution, hypochlorous acid partially dissociates into the anion hypochlorite (OCl^-^):

(2) $$\mathrm{HOCl}\;↔\;H^{+}\;{\mathrm{OCl}}^{–}$$

The ‘available’ chlorine is the sum of the HOCl and OCl^-^ concentrations in the solution.[17] Available chlorine might be defined as a measurement of oxidizing capacity and is expressed in terms of the amount of elemental chlorine. HOCl is considered to be a stronger oxidant than the hypochlorite ion. The HOCl molecule is responsible for the strong chlorinating and oxidizing action on tissue and microorganisms. HOCl dissociation [Equation 2] depends on pH, with the clinical equilibrium between HOCl and OCl^-^ being maintained as HOCl is consumed through its germicidal function.[18] Baker[19] gave the relationship between HOCl, OCl^-^, and pH. At pH 10, basically all chlorine is in the OCl^-^ form; the reverse occurs at a pH of 4.5, when all chlorine is in the form of HOCl. The disinfecting properties decrease with higher pH, paralleling the concentration of dissociated HOCl. Bloomfield and Miles[17] confirmed that hypochlorites at a lower pH possess greater antimicrobial activity. Andrews and Orton[19] reported that HOCl was responsible for the destruction of microorganisms. Morris[20] found that OCl^-^ ion possesses approximately 1/80^th^ of the germicidal potency of HOCl in killing *Escherechia coli*.

Reactive chlorine in aqueous solution at body temperature can take two forms: hypochlorite (OCl^-^) or hypochlorous acid (HOCl). The concentration of these can be expressed as available chlorine by determining the electrochemical equivalent amount of elemental chlorine,[21] according to the following equations:

(1) $${\mathrm{Cl}}_{2}\;+\;2e^{–}\;=\;2{\mathrm{Cl}}^{–}$$

(2) $${\mathrm{OCl}}^{-}\;+\;2e^{–}\;+\;2H^{+}\;=\;{\mathrm{Cl}}^{–}\;+\;H_{2}O$$

Therefore, 1 mol of hypochlorite contains 1 mol of available chlorine. The state of available chlorine is dependant on the pH of the solution. Above a pH of 7.6, the predominant form is hypochlorite and below this value it is hypochlorous acid.[22] Both forms are extremely reactive oxidizing agents. Pure hypochlorite solutions, as are used in endodontics, have a pH of 12,[23] and thus the entire available chlorine is in the form of OCl^-^. However, at identical levels of available chlorine, HOCl is more bactericidal than hypochlorite.[24]

#### 2. Temperature

A rise in temperature by 25°C increased NaOCl efficacy by a factor of 100 (25). The capacity of a 1% NaOCl at 45°C to dissolve human dental pulps was found to be equal to that of a 5.25% solution at 20°C.[26]

#### 3. Ultrasonic

The use of ultrasonic agitation increased the effectiveness of 5% NaOCl in the apical third of the canal wall.[26] Passive ultrasonic irrigation with a nickel-titanium tip produced superior tissue-dissolving effects as compared to sonic irrigant activation.[27]

### Influence on mechanical properties

NaOCl is an efficient organic solvent that causes dentin degeneration because of the dissolution of collagen by the breakdown of the bonds between carbon atoms and disorganization of the proteic primary structure.[28] The reduction of the bond strength seen between adhesive systems and dentin walls may be because of the removal of collagen fibrils from the dentin surface by NaOCl, impeding the formation of a consistent hybrid layer.[29]

### Influence of NaOCl on NiTi

Busslinger and Barbakow[30] evaluated corrosion of endodontic files caused by NaOCl solutions of different concentrations from 0.5% to 5.5%. These authors concluded that the quantities of ions released by the corrosion process into the NaOCl solutions were insignificant. Consequently, no significant corrosion of NiTi files in these solutions was detected. Fabiola *et al*.[31] suggests that exposure to 5.25% NaOCl solution affects neither resistance to flexural fatigue nor torsional resistance of NiTi K3 endodontic files.

### Influence of NaOCl on bond strength

NaOCl irrigation leads to decreased bond strength between dentin and resin cements and may require a reversal agent because of its ability to affect the polymerization of the resin sealer.[32,33] Agents such as ascorbic acid or sodium ascorbate have been shown to completely reverse this reduction in bond strength.[34]

### Interaction of NaOCl and chlorhexidine

Kuruvilla *et al*.[35] suggested that the antimicrobial effect of 2.5% NaOCl and 0.2% chlorhexidine (CHX) used in combination was greater than that of either agent used separately. The reaction between NaOCl and CHX produces a carcinogenic product, parachloroanaline (PCA), the potential leakage of which into the surrounding tissues is a concern. The precipitate is an insoluble neutral salt formed by the acid-base reaction between NaOCl and CHX. PCA is the main product of the interaction of NaOCl and CHX, and has the molecular formula NaC_6_H_4_Cl.[36] When mixed with NaOCl, CHX molecules become hydrolyzed into smaller fragments, each forming a byproduct. The first bonds to be broken in this reaction are those between carbon and nitrogen because of the low-bond dissociation energy between these two atoms. The presence of PCA was confirmed by the Beilstein test for the presence of chlorine and the HCl solubility test for the presence of aniline. Leaching of PCA from the insoluble precipitate formed is of concern because it has been shown to be cytotoxic in rats[37] and possibly carcinogenic in humans.[38–40] This reaction coats the canal surface and significantly occludes the dentinal tubules and affects the seal of the root canal.[41]

## EDTA

EDTA reacts with the calcium ions in dentine and forms soluble calcium chelates. It has been reported that EDTA decalcified dentin to a depth of 20–30 *μ*m in 5 min.[42]

### Time duration for smear layer removal

A continuous rinse with 5 ml of 17% EDTA, as a final rinse for 3 min efficiently removes the smear layer from root canal walls.[43] According to Saito *et al*. greater smear layer removal was found in the 1-min EDTA irrigation group than the 30-sec or 15-sec groups.[44,45]

### Effect on tooth surface strain

Irrigation with 5% NaOCl alone or alternated with 17% EDTA (used in 30-min cycles) significantly increased tooth surface strain. The alternated regimen showed significantly greater changes in tooth surface strain than NaOCl alone. Irrigation with 3% NaOCl and 17% EDTA individually or in combination did not significantly alter the tooth surface strain.[46]

### EDTA with ultrasonics

A 1-min application of 17% EDTA combined with ultrasonics is efficient for smear layer and debris removal in the apical region of the root canal.[47] EDTA performed significantly better than NaCl and NaOCl in smear layer removal and dentinal tubule opening.[48]

### Chlorhexidine

Chlorhexidine digluconate is widely used in disinfection because of its excellent antimicrobial activity. However, it completely lacks tissue dissolving capability.[49]

### Structure and mechanism of action

**Figure 1:**
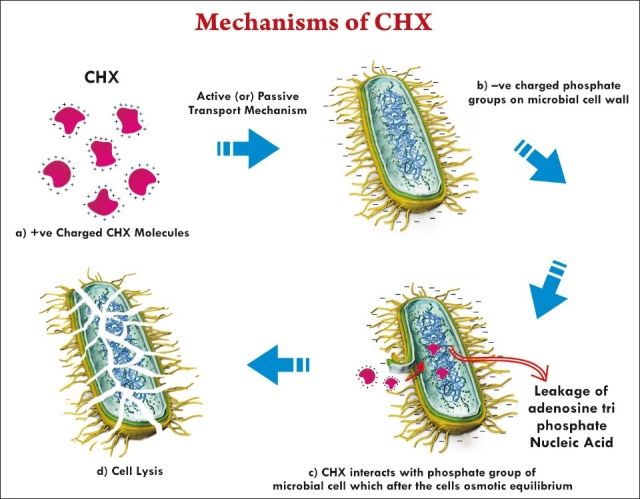
Mechanism of action of CHX.

CHX is a synthetic cationic bis-guanide that consists of two symmetric 4-chlorophenyl rings and two biguanide groups connected by central hexam-ethylene chains.[50] CHX is a positively charged hydrophobic and lipophilic molecule that interacts with phospholipids and lipopolysaccharides on the cell membrane of bacteria and enters the cell through some type of active or passive transport mechanism.[51] Its efficacy is because of the interaction of the positive charge of the molecule with the negatively charged phosphate groups on microbial cell walls,[52,53] which alters the cells’ osmotic equilibrium. This increases the permeability of the cell wall, allowing the CHX molecule to penetrate into the bacteria.[49] Damage to this delicate membrane is followed by leakage of intracellular constituents, particularly phosphate entities such as adenosine triphosphate and nucleic acids. As a consequence, the cytoplasm becomes congealed, with resultant reduction in leakage; thus, there is a biphasic effect on membrane permeability. CHX antimicrobial activity is pH dependant, with the optimal range being 5.5–0.7 [Figure 1].[54]

### Antibacterial activity

Basson and Tait[55] compared the ex vivo effectiveness of calcium hydroxide, iodine potassium iodide (IKI), and CHX solution in disinfecting root canal systems that were infected with *Actinomyces israelii*. The root canals were exposed to either IKI, calcium hydroxide, or 2% CHX for periods of 3, 7, and 60 days. CHX was the only disinfectant that was able to eliminate *A israelii*. Oncag *et al*.[56] evaluated the antibacterial properties against *Enterococcus faecalis* of 5.25% NaOCl, 2% CHX, and 0.2% CHX plus 0.2% cetrimide after 5 min and 48 h. The 2% CHX and Cetrexidin^®^ were significantly more effective against E faecalis. Two studies[57,58] have investigated the antimicrobial activity against endodontic pathogens of three concentrations (0.2%, 1%, and 2%) of two forms of CHX (gel and liquid) and compared them with five concentrations of NaOCl (0.5%, 1%, 2.5%, 4%, and 5.25%). Both the 2% gel and 2% liquid formulations of CHX eliminated *Staphylococcus aureus* and *Candida albicans* within 15 sec, whereas the gel formulation killed *E faecalis* within 1 min. All of the tested irrigants eliminated *Porphyromonas endodontalis, Porphyromonas gingivalis*, and *Prevotella intermedia* within 15 sec.[57,58]

### Effect of CHX on dentin

CHX has the ability to bind anionic molecules such as phosphate present in the structure of hydroxyapatite. Phosphate exists in calcium carbonate complexes in dentin. CHX can bind phosphate, which leads to release of small amounts of calcium from the root canal dentin.[60]

### Interaction of CHX and EDTA

When CHX and EDTA interact, a precipitate is formed that is over 90% CHX and EDTA, with less than 1% of the potential decomposition product, p-chloroaniline. The high recovery indicates that CHX is not degraded by EDTA under normal conditions. The precipitate is most likely a salt formed by electrostatic neutralization of cationic CHX by anionic EDTA. The suspected net ionic equation is:

$$2{\mathrm{HEDTA}}_{\left(\mathrm{aq}\right)}^{3-}\;+\;3H_{2}{\mathrm{CHX}}_{\left(\mathrm{aq}\right)}^{2+}\;↔\;{\left(\mathrm{HEDTA}\right)}_{2}\;{\left(H_{2}\mathrm{CHX}\right)}_{3\left(s\right).}\;$$

The clinical significance of this precipitate is largely unknown.[60]

### CHX and bioflim

Spratt *et al*. have evaluated the effectiveness of 2.25% NaOCl, 0.2% CHX, 10% povidone iodine against monoculture biofilms of *P intermedia, P miros, S intermedius, F nucleatum*, and *E faecalis*. They reported that NaOCl was the most effective antimicrobial agent, followed by the iodine solution.[61] Clegg *et al*. evaluated the ex vivo effectiveness against apical dentine biofilms of three concentrations of NaOCl (6%, 3%, and 1%), 2% CHX, and Mixture of Tetracycline acid and detergents (MTAD). They reported that the 6% NaOCl and 3% NaOCl were capable of disrupting and removing the biofilm, the 1% NaOCl and the MTAD were capable of disrupting the biofilm but did not eliminate the bacteria, and the 2% CHX was not capable of disrupting the biofilm.[62]

### Substantivity

White *et al*. evaluated the antimicrobial substantivity of a 2% CHX solution as an endodontic irrigant and reported that the substantivity lasted 72 h.[63] Khademi *et al*.[64] found that 5-min application of 2% CHX solution induced substantivity for up to 4 weeks. Rosenthal *et al*.[65] evaluated the substantivity of 2% CHX solution within the root canal system after 10 min of application and they reported that the CHX was retained in the root canal dentine in antimicrobially effective amounts for up to 12 weeks. Antimicrobial substantivity depends on the number of CHX molecules available to interact with the dentine.[49]

### CHX and dentine bonding (anticollagenolytic activity)

Human dentin contains at least collagenase (MMP-8), gelatinases MMP-2 and MMP-9, and enamelysin MMP-20.[66,67] Dentine collagenolytic[68] and gelatinolytic activities[68] can be suppressed by protease inhibitors, indicating that MMP inhibition could be beneficial in the preservation of hybrid layers. This was demonstrated in an *In vivo* study in which the application of CHX, known to have a broad-spectrum MMP-inhibitory effect,[69] significantly improved the integrity of the hybrid layer in a 6-month clinical trial.[70] Auto-degradation of collagen matrices can occur in resin-infiltrated dentine but may be prevented by the application of a synthetic protease inhibitor such as CHX.[71] On the whole, because of its broad-spectrum MMP-inhibitory effect, CHX can significantly improve the resin–dentine bond stability.

### Cytotoxicity of CHX

Cytotoxic effects of CHX on canine embryonic fibroblast and *Staphylococcus aureus* showed that bactericidal concentrations were lethal to canine embryonic fibroblasts while non-cytotoxic concentrations allowed survival of bacteria.[72] Ribeiro *et al*.[73] evaluated the genotoxicity (potential damage to DNA) of formocresol, paramonochlorophenol, calcium hydroxide, and CHX against Chinese hamster ovary cells. Results showed that none of the mentioned agents contributed to DNA damage. Thus, in the clinically used concentrations, the biocompatibility of CHX is acceptable.

### Allergic reactions to CHX

Contant dermatitis is a common adverse reaction.[74] CHX may have a number of rare side effects, such as desquamative gingivitis, discoloration of the teeth and tongue, or dysgeusia.[49]

## MTAD

Torabinejad *et al*. developed a irrigant with combined chelating and antibacterial properties.[75] MTAD is a mixture of 3% doxycycline, 4.25% citric acid, and detergent (Tween-80).[75,76]

### Antibacterial activity and smear layer removal

MTAD is composed of three constituents that are expected to act synergistically against bacteria.[75] The bactericidal effect of MTAD was inferior to 1%-6% NaOCl against *E faecalis* biofilms.[77] The antibacterial activity of MTAD might also be inhibited by the buffering effect of dentin and the serum albumin present in the root canal.[78] MTAD has been reported to be effective in removing smear layer.[79] In the MTAD preparation, the citric acid may serve to remove the smear layer, allowing doxycycline to enter the dentinal tubules and exert an antibacterial effect.[80] The recently revised protocol for clinical use of MTAD advises an initial irrigation for 20 min with 1.3% NaOCl, followed by a 5-min final rinse with MTAD.[80]

### Bond strength

The use of MTAD as a final rinse with gutta-percha/AH Plus^®^ resulted in a significant reduction in bond strength (1.76±1.67 Mpa) when compared with EDTA.[81] A final rinse with MTAD might have a negative effect on the bonding ability of both resin-based and calcium hydroxide–based sealers due to the precipitate formation.[82]

## OTHER IRRIGANTS

### Citric acid and EDTA-T

The use of 10% citric acid as final irrigation has shown good results in smear layer removal.[83] *In vitro* studies have shown their cytotoxicity, and 10% citric acid has proven to be more biocompatible than 17% EDTA-T and 17% EDTA.[84,85].

Scelza *et al* evaluated the inflammatory response of 17% EDTA, 17% EDTA-T, and 10% citric acid in bony defect created in rat jaws and they concluded that 10% citric acid showed less aggressive in inflammatory response.[86] The use of 25% citric acid was found to be ineffective in eradication of biofilms of *E faecalis* after 1, 5, and 10 min of exposure.[87]

### Maleic acid

Maleic acid is a mild organic acid used as an acid conditioner in adhesive dentistry.[89] Ballal *et al*. reported that final irrigation with 7% maleic acid for 1 min was more efficient than 17% EDTA in the removal of smear layer from the apical third of the root canal system.[89]

## HEBP

HEBP (1-hydroxyethylidene- 1, 1-bisphosphonate), also known as etidronic acid or etidronate, has been proposed as a potential alternative to EDTA or citric acid because this agent shows no short-term reactivity with NaOCl.[90] HEBP is nontoxic and has been systematically applied to treat bone diseases.[91] The demineralization kinetics promoted by both 9% HEBP and 18% HEBP were significantly slower than those of 17% EDTA.[92] De-Deus *et al*. reported that the soft chelating irrigation protocol (18% HEBP) optimized the bonding quality (3.1–6.1 MPa) of Resilon/Epiphany^®^.[93]

### Chlorine dioxide

Chlorine dioxide (ClO _2_) is chemically similar to chlorine or hypochlorite, the familiar household bleach. An In vitro study compared organic tissue dissolution capacity of NaOCl and ClO_2_. It was concluded that ClO_2_ and NaOCl are equally efficient for dissolving organic tissue.[94] ClO_2_ produces little or no trihalomethanes.[95] A study showed that trihalomethane is an animal carcinogen and a suspected human carcinogen.[96] ClO_2_ might therefore be a better dental irrigant than NaOCl.[97]

### Silver diamine fluoride

A 3.8% w/v silver diamine fluoride (Ag[NH_3_]_2_F) solution has been developed for intracanal irrigation. This represents a 1:10 dilution of the original 38% Ag(NH_3_)_2_ F solution used for root canal infection.[98] The study on the antibacterial effect of 3.8% Ag(NH_3_)_2_F against a *E faecalis* biofilm model concluded that Ag(NH_3_)_2_F has potential for use as an antimicrobial root canal irrigant or interappointment medicament to reduce bacterial loads.[99] *E faecalis* was completely killed by Ag(NH^3^)^2^F after exposure to these agents for 60 min. The silver deposits were found to occlude tubular orifices after removal of the smear layer.

### Tetraclean^®^

Tetraclean is a mixture of doxycycline hyclate (at a lower concentration than in MTAD), an acid, and a detergent.[100,101] It is able to eliminate microorganisms and smear layer in dentinal tubules of infected root canals with a final 5-min rinse. Comparison of antimicrobial efficacy of 5.25% NaOCl, MTAD, and Tetraclean^®^ against *E faecalis* biofilm showed that only 5.25% NaOCl could consistently disgregate and remove the biofilm at every time interval. However, treatment with Tetraclean^®^caused a high degree of biofilm disgregation in every considered time interval (5, 30, and 60 min at 20°C) as compared with MTAD.[102]

### Triclosan and Gantrez^®^

Triclosan is a broad spectrum antimicrobial agent, active against gram-positive and gram-negative bacteria as well as some fungi and viruses.[103,104] Nudera *et al*.[105] evaluated the minimum inhibitory concentrations (MIC) and minimum bactericidal concentrations (MBC) of triclosan and triclosan with Gantrez^®^ against *P intermedia, F nucleatum, A naeslundii, P gingivalis,* and *E faecalis*. The MBC of triclosan ranged from 12-94 μg/ml. The MBC of triclosan with Gantrez^®^ ranged from <0.3-10.4 μg/ml. The addition of Gantrez^®^ enhanced the bactericidal activity of triclosan. Both triclosan and triclosan with Gantrez^®^ demonstrated bactericidal activity against the five specific endodontic pathogens.

### Herbal

#### Triphala

*Triphala* consists of dried and powdered fruits of three medicinal plants *Terminalia bellerica, Terminalia chebula*, and *Emblica officinalis*.[106] *Triphala* achieved 100% killing of *E faecalis* at 6 min. This may be attributed to its formulation, which contains three different medicinal plants in equal proportions; in such formulations, different compounds may help enhance the potency of the active compounds, producing an additive or synergistic effect.[107] Triphala contains fruits that are rich in citric acid, which may aid in removal of the smear layer. The major advantages of using herbal alternatives are easy availability, cost-effectiveness, longer shelf life, low toxicity, and lack of microbial resistance.[108]

#### Green tea

Green tea polyphenols, the traditional drink of Japan and China is prepared from the young shoots of the tea plant *Camellia sinensis*.[109] Green tea polyphenols showed statistically significant antibacterial activity against *E faecalis* biofilm formed on tooth substrate. It takes 6 min to achieve 100% killing of *E faecalis*.[107]

#### Morinda citrifolia

Morinda citrifolia (MCJ) has a broad range of therapeutic effects, including antibacterial, antiviral, antifungal, antitumor, antihelmintic, analgesic, hypotensive, anti-inflammatory, and immune-enhancing effects.[110–113] MCJ contains the antibacterial compounds L-asperuloside and alizarin[113]. Murray *et al*.[113] proved that, as an intracanal irrigant to remove the smearlayer, the efficacy of 6% MJC was similar to that of 6% NaOCl in conjunction with EDTA. The use of MCJ as an irrigant might be advantageous because it is a biocompatible antioxidant[113] and not likely to cause severe injuries to patients as might occur through NaOCl accidents.

## CONCLUSION

During instrumentation canals should be irrigated using copious amounts of the NaOCl solution. Once the shaping procedure is completed, canals can be thoroughly rinsed using aqueous EDTA or citric acid. Generally each canal is rinsed for at least 1 min using 5 to 10 ml of the chelator irrigant. After the smear layer removal procedure, a final rinse with an antiseptic solution appears beneficial. Chlorhexidine appears to be the most promising agent for use as a final irrigant in this situation. It has an affinity for dental hard tissues and, once bound to a surface, it has prolonged antimicrobial activity, a phenomenon called substantivity. After the introduction of MTAD irrigant, newer irrigating regimen followed was initial rinse with 1.3 % NaOCl for 20 min and followed by final rinse with MTAD for 5 min. Future research on irrigants needs to focus on finding a single irrigant that has tissue dissolving capacity, smear layer removal property, and antibacterial efficacy.
